# Incidental Findings of Muscle-Origin Calcifications in the Sternocleidomastoid Muscle on an Orthopantomogram

**DOI:** 10.7759/cureus.80016

**Published:** 2025-03-04

**Authors:** Francesco Valente, Riccardo Riccardi, Pierluigi Valente, Roberto Del Rosso, Andrea Sbrenna

**Affiliations:** 1 Department of Dentistry, San Damiano Dental Clinic, Rome, ITA; 2 School of Dentistry, San Raffaele University, Milan, ITA; 3 Department of Dentistry, Humanis Dental Center, Perugia, ITA

**Keywords:** dental orthopantomogram, dental panoramic x-ray, muscle origin calcifications, sternocleidomastoid, ultrasound

## Abstract

Calcifications of muscular origin are rare findings in dental panoramic radiographs and can pose diagnostic challenges if not properly identified. This case report describes the incidental discovery of bilateral calcifications within the sternocleidomastoid (SCM) muscle on a routine dental panoramic radiograph. A comprehensive differential diagnosis was considered, including trauma, inflammation, metabolic disorders, and idiopathic conditions. The absence of systemic disorders or recent trauma supported a diagnosis of dystrophic calcifications of the SCM muscle. This case underscores the importance of evaluating anatomical structures beyond the dental and bony regions in panoramic radiography to avoid misdiagnosis.

## Introduction

Dental panoramic radiographs are commonly used in dentistry for evaluating dental and bony structures. Occasionally, they reveal incidental findings unrelated to the dental condition being examined. Muscle calcifications are incidental findings in radiological examinations [1-2]. While they are generally asymptomatic, their presence may raise concerns for the treating clinician [3]. The sternocleidomastoid (SCM) muscle is a prominent neck muscle that plays a crucial role in head and neck movement. The SCM has two heads. The sternal head originates from the manubrium of the sternum. The clavicular head originates from the medial portion of the clavicle. Both heads are inserted at the mastoid process of the temporal bone and the superior nuchal line of the occipital bone [4]. The cranial part of the SCM muscle is more likely to be visualized on a dental panoramic radiograph. Specifically, the area where the SCM muscle inserts into the mastoid process of the temporal bone may overlap with the mandible, particularly the ramus and angle, and therefore appear on the radiograph. Panoramic radiographs extend their field of view beyond the teeth and mandible to include adjacent soft tissues and skeletal structures in the neck and skull base. Because the mastoid process is anatomically close to the mandible, any calcifications or changes in the cranial SCM may be visualized as overlapping or incidental findings, potentially causing diagnostic confusion.

This article was previously posted to the Research Square preprint server on January 15, 2025.

## Case presentation

**Figure 1:**
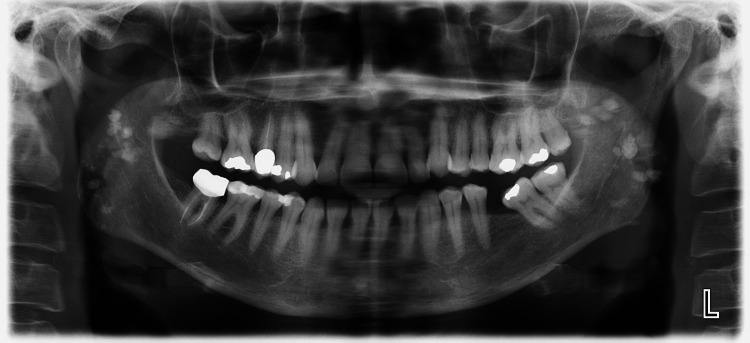
Panoramic radiograph showing calcifications in the sternocleidomastoid muscle.

A 59-year-old female patient presented for a routine dental examination at our clinic, San Damiano Dental Center, in Rome, Italy, in July 2023. She reported no significant medical history, systemic conditions, or previous head and neck trauma. No symptoms such as pain, swelling, or restricted jaw movement were noted. As part of the routine evaluation, a panoramic radiograph (orthopantomogram) was obtained. The imaging revealed multiple small, well-defined radiopaque structures bilaterally in the region of the mandibular ramus, superimposed over the osseous structures. These findings were incidental and did not correlate with any clinical signs or symptoms (Figure 1).

**Figure 2:**
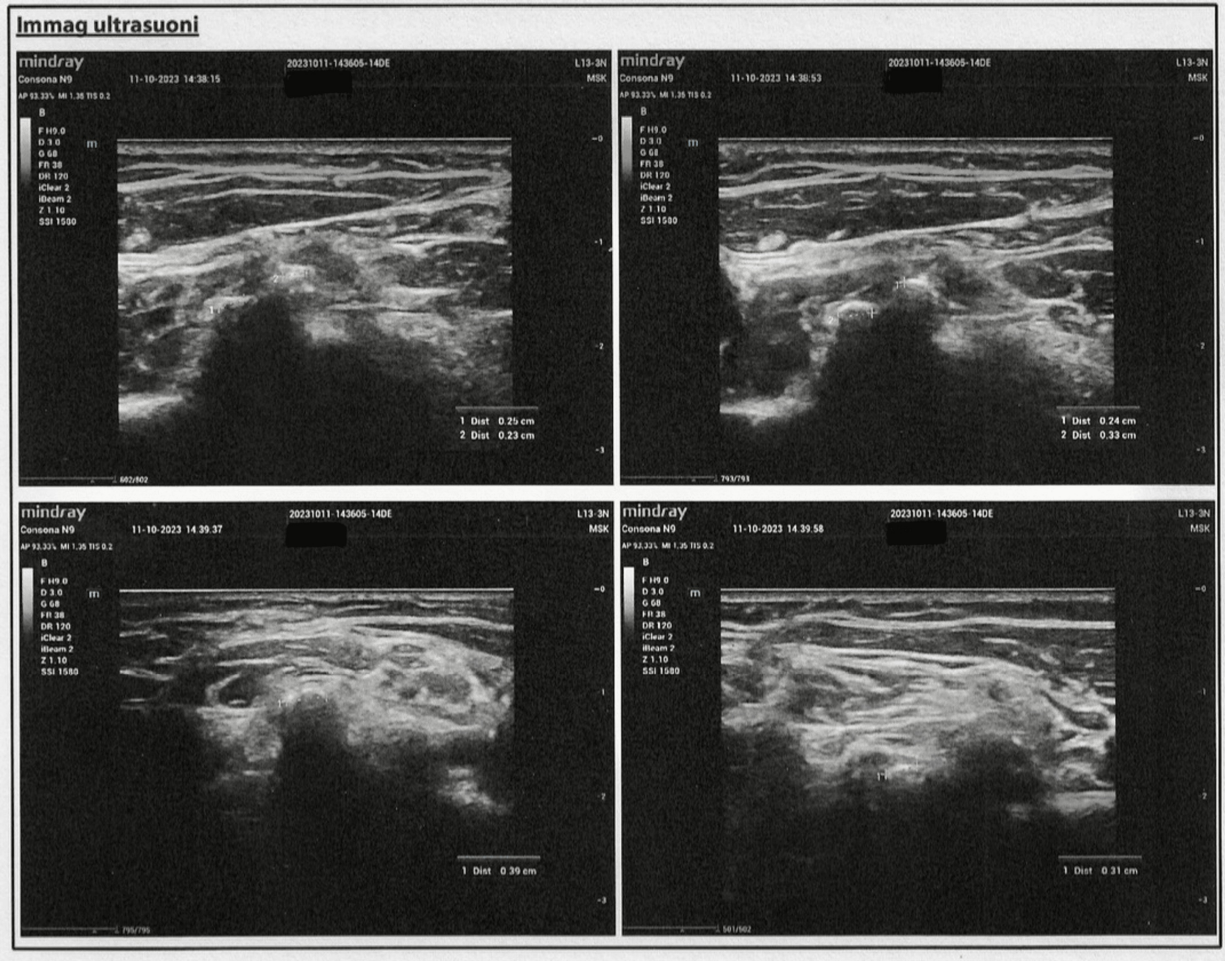
Ultrasound imaging of multiple small calcified deposits within the muscle belly of the sternocleidomastoid on both sides.

To further investigate the nature and exact localization of these calcifications, an ultrasound examination was performed. Ultrasound is a valuable imaging modality for assessing soft tissue calcifications without exposing the patient to additional radiation. The ultrasound evaluation revealed multiple small calcific deposits located within the muscle belly of the SCM muscle on both sides. The dimensions of the calcifications varied from 2 mm to 6 mm in diameter. No associated hematomas, myofascial discontinuities, or signs of acute inflammation were observed. Additionally, the main vascular axes of the neck and the major salivary glands appeared normal bilaterally, ruling out vascular or glandular involvement (Figure 2).

To further characterize the calcifications and exclude other potential differential diagnoses, a cone-beam computed tomography (CBCT) scan was considered. However, given the absence of clinical symptoms and the clear visualization provided by ultrasound, additional imaging was deemed unnecessary at this stage. Given the absence of systemic disorders, prior trauma, or inflammatory conditions, the calcifications were classified as dystrophic muscle calcifications. The patient was informed of the findings and reassured regarding their benign nature. Since no symptoms were present, no further intervention was deemed necessary. However, follow-up was recommended to monitor for any potential changes over time. As of the date this article was submitted, the patient is still under follow-up. No symptoms have been observed, and no progression in the size or number of the calcifications has been noted during this period. Written informed consent for publication was obtained from the patient referenced in this article. The research is based solely on imaging data collected from routine diagnostic procedures, with no additional data collection or patient interaction involved.

## Discussion

Calcifications in the SCM muscle are rarely addressed in dental scientific literature, making this case noteworthy [5-6]. While several publications discuss calcifications in the masseter and other masticatory muscles [7], there is a clear gap concerning calcifications in the SCM muscle [8-9]. This highlights the importance of recognizing such findings in panoramic radiographs, as they can be misinterpreted without a proper understanding of overlapping anatomical structures [10-11]. Previous studies have documented muscle-origin calcifications resulting from trauma, chronic inflammation, or genetic predispositions [12]. The exact mechanism of their formation remains unclear, but it is thought to be related to tissue injury and subsequent repair processes. Muscle calcifications can also occur in conditions such as fibrodysplasia ossificans progressiva (FOP), a rare genetic disorder characterized by progressive heterotopic ossification, leading to significant functional impairment [13]. Muscle-origin calcifications, particularly in the SCM, are uncommon in routine panoramic imaging. Their etiology varies, and they are traditionally classified into dystrophic, iatrogenic, metastatic, and idiopathic categories based on the mechanism of formation and clinical correlation. Alternatively, a classification based on anatomical compartment (subcutaneous, neurovascular, fascial, muscle, periarticular) can aid in the radiological assessment.

Dystrophic and iatrogenic calcifications

These occur in damaged or degenerating tissue and account for 95-98% of all soft-tissue calcifications. They typically arise due to chronic inflammation, trauma, surgical manipulation, or medication infusion.

Metastatic calcifications

These result from calcium-phosphate metabolism imbalances, often due to hyperparathyroidism, chronic kidney disease, or other systemic disorders. Although they typically affect high-blood-flow areas (lungs, kidneys, vessels), they can also appear in muscles. In this case, the patient had no systemic illnesses or metabolic disorders, making metastatic calcification unlikely.

Idiopathic calcifications

These include conditions such as tumoral calcinosis, a rare hereditary disorder affecting phosphate metabolism. It often presents with periarticular calcified masses, resembling metastatic calcifications, primarily on bursal surfaces of major joints [14].

Infectious and traumatic causes

Muscle calcifications can result from infectious processes such as granulomatous diseases and parasitic infections, including cysticercosis [15]. Traumatic causes include myositis ossificans, which leads to heterotopic ossification after injury. Despite its name, myositis ossificans lacks an inflammatory component, involving only bone formation rather than muscle inflammation. Other traumatic causes include calcified muscle hematomas and calcific myonecrosis.

During routine dental panoramic radiography, the most common radiopaque lesions overlapping the mandibular ramus are tonsilloliths [16]. These calcified deposits form in the palatine tonsil crypts and appear as radiopaque masses near the ascending mandibular ramus, making differentiation from muscle-origin calcifications difficult [17]. In this case, tonsilloliths were initially considered, given their anatomical location and frequent occurrence. However, the calcifications extended beyond the tonsillar fossa and the ascending mandibular ramus, ruling out this diagnosis. Another important differential diagnosis is myositis ossificans, characterized by abnormal bone formation within muscle tissue following trauma. Imaging features typically include well-defined radiopacity within the muscle belly, with progressive calcification on serial imaging.

Phleboliths may also appear radiographically in the region of the mandibular ramus; however, these lesions exhibit a highly pathognomonic appearance, often resembling a round (spherical), well-defined, target-like structure with a radiopaque center surrounded by a radiolucent halo, and this highly pathognomonic feature makes it virtually impossible to confuse them with tonsilloliths, muscle calcifications, or myositis ossificans [18].

The superimposition of soft tissue calcifications over osseous structures in panoramic radiographs can cause diagnostic ambiguity. Because dental panoramic radiographs are two-dimensional, calcifications from various anatomical sites may be mistakenly associated with the jawbones. In this patient, the absence of trauma or systemic inflammation complicated the determination of etiology. Ultrasound played a crucial role in localizing the calcifications within the SCM muscle without additional radiation exposure [19].

Given the benign nature of the lesions and the absence of symptoms, a biopsy was deemed unnecessary, while the absence of a biopsy or advanced imaging (i.e., CBCT or MRI) may be viewed as a limitation; ultrasound was chosen to minimize radiation exposure, which is particularly important in dental imaging. In cases where diagnosis remains unclear, additional imaging such as CBCT or MRI can provide detailed views of soft tissue structures and improve calcification localization.

## Conclusions

This case report highlights the importance of a comprehensive clinical and imaging evaluation when assessing incidental findings on panoramic radiographs. The calcifications detected in this patient were localized within the SCM muscle, reinforcing the necessity of considering overlapping anatomical structures to prevent misdiagnosis. Given that panoramic radiography is a two-dimensional imaging modality, it often presents diagnostic challenges due to superimposition. Clinicians should remain vigilant when encountering unexpected radiopaque findings on panoramic radiographs, considering a broad differential diagnosis that includes muscle-origin calcifications. Future studies could further investigate the prevalence, etiology, and clinical implications of such calcifications, providing additional insights into their significance in dental practice.

## References

[1] Katz JO, Langlais RP, Underhill TE, Kimura K (1989). Localization of paraoral soft tissue calcifications: the known object rule. Oral Surg Oral Med Oral Pathol.

[2] Monsour PA, Mendoza AR (1990). Panoramic ghost images as an aid in the localization of soft tissue calcifications. Oral Surg Oral Med Oral Pathol.

[3] Banks KP, Bui-Mansfield LT, Chew FS, Collinson F (2005). A compartmental approach to the radiographic evaluation of soft-tissue calcifications. Semin Roentgenol.

[4] Bordoni B, Jozsa F, Varacallo MA (2025). Anatomy, head and neck, sternocleidomastoid muscle. StatPearls [Internet].

[5] de Faria LL, Babler F, Ferreira LC, de Noronha Junior OA, Marsolla FL, Ferreira DL (2020). Soft tissue calcifications: a pictorial essay. Radiol Bras.

[6] Steiner M, Gould AR, Kushner GM, Lutchka B, Flint R (1997). Myositis ossificans traumatica of the masseter muscle: review of the literature and report of two additional cases. Oral Surg Oral Med Oral Pathol Oral Radiol Endod.

[7] Kim HY, Park JH, Lee JB, Kim SJ (2017). A case of dystrophic calcification in the masseter muscle. Maxillofac Plast Reconstr Surg.

[8] Karaali S, Emekli U (2018). Myositis ossificans traumatica of the medial pterygoid muscle after third molar tooth extraction: a case report and review of literature. J Oral Maxillofac Surg.

[9] Lello GE, Makek M (1986). Traumatic myositis ossificans in masticatory muscles. J Maxillofac Surg.

[10] Patel S, Richards A, Trehan R, Railton GT (2008). Post-traumatic myositis ossificans of the sternocleidomastoid following fracture of the clavicle: a case report. Cases J.

[11] Hanisch M, Hanisch L, Fröhlich LF, Werkmeister R, Bohner L, Kleinheinz J (2018). Myositis ossificans traumatica of the masticatory muscles: etiology, diagnosis and treatment. Head Face Med.

[12] Pignolo RJ, Shore EM, Kaplan FS (2013). Fibrodysplasia ossificans progressiva: diagnosis, management, and therapeutic horizons. Pediatr Endocrinol Rev.

[13] Önal M, Bajin MD, Yılmaz T (2014). Fibrodysplasia ossificans progressiva: a case report. Turk J Pediatr.

[14] Olsen KM, Chew FS (2006). Tumoral calcinosis: pearls, polemics, and alternative possibilities. Radiographics.

[15] Lal T, Paramasivam S, Jayapal B, Kataria R (2021). Solitary cysticercosis of the sternocleidomastoid muscle. BMJ Case Rep.

[16] Takahashi A, Sugawara C, Kudoh T (2017). Prevalence and imaging characteristics of palatine tonsilloliths evaluated on 2244 pairs of panoramic radiographs and CT images. Clin Oral Investig.

[17] Babu BB, Tejasvi MLA, Avinash CK, B C (2013). Tonsillolith: a panoramic radiograph presentation. J Clin Diagn Res.

[18] Abrantes TC, Barra SG, Silva LV, Abrahão AC, Mesquita RA, Abreu LG (2022). Phleboliths of the head and neck region - a case report. Ann Maxillofac Surg.

[19] Özkan G, Köse E, Yeşiltepe S (2023). ultrasonographic evaluation of soft tissue calcifications in the head and neck region detected on panoramic radiographs. J Ultrasound Med.

